# 5α,6α-Ep­oxy-7-norcholestan-3β-yl acetate

**DOI:** 10.1107/S1600536811054249

**Published:** 2011-12-23

**Authors:** L. C. R. Andrade, J. A. Paixão, M. J. M. de Almeida, J. F. S. Carvalho, M. M. Cruz Silva

**Affiliations:** a CEMDRX, Department of Physics, Faculty of Sciences and Technology, University of Coimbra, P-3004-516 Coimbra, Portugal; b Centre for Neuroscience and Cell Biology, University of Coimbra, P-3004-517 Coimbra, Portugal; c Faculty of Pharmacy, University of Coimbra, P-3000-548 Coimbra, Portugal

## Abstract

The title cholestan, C_28_H_46_O_3_, was prepared by epoxidation of 7-norcholest-5-en-3β-yl acetate and crystallized by slow evaporation from an ethano­lic solution. All rings are *trans* fused. The 3β-acetate and the 17β-cholestane side chain are in equatorial positions. The mol­ecule is highly twisted due to its B-nor characteristic. A quantum chemical *ab-initio* Roothaan Hartree–Fock calculation of the equilibrium geometry of the isolated mol­ecule gives values for bond lengths and valency angles in close agreement with the experimental ones.

## Related literature

For the chemistry of the title compound, see: Carvalho *et al.* (2009*a* ▶, 2010*a* ▶). For studies of biological activity of steroids, see: Carvalho *et al.* (2009*b* ▶, 2010*b* ▶). For the influence of structural characteristics of B-nor steroids on the outcome of many reactions, see: Uyanik & Hanson (2009) ▶. For asymmetry parameters, see: Duax & Norton (1975 ▶); Altona *et al.* (1968 ▶). For reference bond-length data, see: Allen *et al.* (1987 ▶). For puckering parameters, see: Cremer & Pople (1975 ▶). For the melting point of the title compound, see: Joska *et al.* (1963 ▶). For the software used in *ab-initio* calculations, see Schmidt *et al.* (1993 ▶).
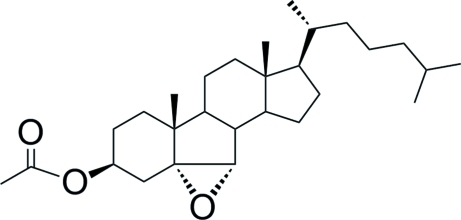


## Experimental

### 

#### Crystal data

C_28_H_46_O_3_
*M* *_r_* = 430.65
Monoclinic, 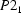

*a* = 7.5820 (1) Å
*b* = 9.7487 (1) Å
*c* = 17.8588 (2) Å
β = 93.1792 (18)°
*V* = 1318.00 (3) Å^3^
*Z* = 2
Mo *K*α radiation
μ = 0.07 mm^−1^
*T* = 293 K
0.44 × 0.24 × 0.16 mm

#### Data collection

Bruker APEX CCD area-detector diffractometer
Absorption correction: multi-scan (*SADABS*; Sheldrick, 2000 ▶) *T*_min_ = 0.981, *T*_max_ = 0.989
35109 measured reflections
6194 independent reflections
3551 reflections with *I* > 2σ(*I*)
*R*_int_ = 0.030

#### Refinement

*R*[*F* ^2^ > 2σ(*F* ^2^)] = 0.048
*wR*(*F* ^2^) = 0.138
*S* = 0.96
6194 reflections
286 parameters
1 restraint
H-atom parameters constrained
Δρ_max_ = 0.23 e Å^−3^
Δρ_min_ = −0.14 e Å^−3^
Absolute structure: Flack (1983 ▶), 2846 Friedel pairs
Flack parameter: −0.5 (15)

### 

Data collection: *SMART* (Bruker, 2003 ▶); cell refinement: *SAINT* (Bruker, 2003 ▶); data reduction: *SAINT*; program(s) used to solve structure: *SHELXS97* (Sheldrick, 2008 ▶); program(s) used to refine structure: *SHELXL97* (Sheldrick, 2008 ▶); molecular graphics: *PLATON* (Spek, 2009) ▶; software used to prepare material for publication: *SHELXL97*.

## Supplementary Material

Crystal structure: contains datablock(s) global, I. DOI: 10.1107/S1600536811054249/im2347sup1.cif

Structure factors: contains datablock(s) I. DOI: 10.1107/S1600536811054249/im2347Isup2.hkl

Additional supplementary materials:  crystallographic information; 3D view; checkCIF report

## Figures and Tables

**Fig. 1.**
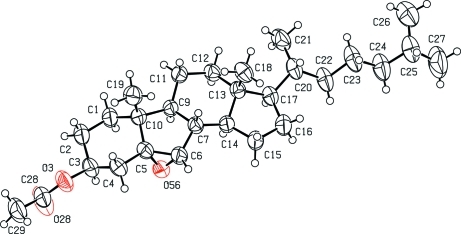
ORTEPII plot of the title compound. Displacement ellipsoids are drawn at the 50% level.

## supplementary crystallographic information

### Comment

Within our interest on the chemistry (Carvalho *et al.*, 2009*a*, Carvalho *et al.*, 2010*a*) and biological activity (Carvalho *et al.*, 2009*b*, Carvalho *et al.*, 2010*b*) of steroids, we have been exploring the cytotoxic potential of oxysterols and their synthetic analogues against a panel of cancer and normal cell lines. We found that several chemical features are important for cytotoxicity such as the cholestane side-chain, a free or an *in vivo* easily generated 3β-hydroxyl group and the presence of an additional hydroxyl group at either 6β- or 7β-position. B-Norsteroids comprise a particular class of steroids, which bear a five-membered ring B, instead of the usual six-membered ring. As discussed recently (Uyanik & Hanson, 2009) this structural characteristic affects the outcome of many reactions, and quite often unexpected products are obtained. On the other hand, very few studies address the consequences of a shorter ring B in biological outcome comparatively to the normal six-membered analogs. We have recently found that an α-epoxide in position C5 and C6 of a cholestane affords different cytotoxic results, when ring B bears only five carbons, instead of the usual six-membered ring B of cholesterol. Specifically, the B-nor-α-epoxycholestane displays a higher cytotoxicity (IC_50 _= 40.9 µ*M*) than the six-membered ring B analogue (IC_50 _> 65 µ*M*) (Carvalho *et al.*, 2009*b*). We hypothesized that such biological result must be correlated with the α-orientation of the two epoxycholestane derivates. In this way, the evaluation of the three-dimensional structure of compound (I) by X-ray crystallography will contribute to correlate the importance of the geometry of ring B and the orientation of the epoxy moiety with the biological effect observed. Single crystal diffraction measurements allowed us to conclude that ring bond lengths have normal values (Allen *et al.*,1987) with average C(*sp*^3^)–C(*sp*^3^) of 1.530 (3) Å, excluding the shorter C5–C6 bond of 1.458 (3) Å. The cholestane side-chain shows C(*sp*^3^)–C(*sp*^3^) bond lengths varying from C24–C25 [1.480 (4) Å] up to C17–C20 [1.540 (3) Å]. Rings A and C have slightly flattened chair conformations. The five membered ring B assumes a 9α-envelope conformation [pseudo-rotation (Altona *et al.*, 1968; Duax & Norton, 1975): ΔC_s_(9)=3.0 (3), ΔC_s_(7)=24.8 (3); ΔC_2_(7,9)=15.0 (3); Δ=173.0 (8); φ=37.9 (2)°; puckering parameters (Cremer & Pople, 1975) q_2_=0.374 (3) Å and φ_2_=285.2 (5)°]. Ring D has a 13β,14α-half chair conformation [ΔC_2_(13,14)=3.4 (3), ΔC_s_(14)=20.0 (3); ΔC_s_(13)=15.8 (3); Δ=5.0 (6); φ=47.2 (2)°; q_2_=0.465 (3) Å and φ_2_=194.9 (4)°]. A pseudo-torsion C19–C10···C13–C18 angle of 12.68 (18)° indicates that, due to the B-nor characteristic, the molecule is highly twisted. Both the 3β-acetate and the 17β-cholestane side-chain are in equatorial positions with angles 66.5 (2) and 65.6 (2)°, respectively. The 5α,6α epoxy plane makes an angle of 83.93 (14)° with the five membered B ring.

In order to gain some insight on how the crystal packing of (I) might affect the molecular geometry we have performed Hartree-Fock quantum chemical calculation using the GAMESS code (Schmidt *et al.*, 1993), of the equilibrium geometry for the free molecule.

These *ab-initio* calculations reproduce well the observed bond lengths and valency angles of the molecule with the exception of a few C–C bonds in the cholestane ligand that are somewhat larger than the measured values, probably as a result of the larger displacement ellipsoids of these atoms. The calculation also reproduces the observed molecular conformation, with puckering parameters that agree well with those determined from the crystallographic study. The high value of the pseudo-torsion angle is well reproduced by the calculations (obs: 12.68 (18), calc: 13.5°). The calculated configuration of the 3β-acetate and the 17β-cholestane side-chain are also close to those observed in the crystal.

Since there is no strong hydrogen bond donor in the molecule, cohesion of the crystal structure can only be attributed to van der Waals interactions.

### Experimental

Synthesis of (I) was performed as described in the literature (Carvalho *et al.*, 2009*a*). Epoxidation of 7-norcholest-5-en-3β-yl acetate in acetonitrile at reflux temperature affords the α-epoxide in high yield (around 90%) in 10 minutes. Crystallization was performed at room temperature by slow evaporation from an ethanolic solution. Mp 386–386.5 K (EtOH); lit., (Joska *et al.*, 1963) 384–385 K.

### Refinement

All hydrogen atoms were refined as riding on their parent atoms using *SHELXL97* defaults. Number of Friedel pairs measured: 2848 (85%). Due to the lack of any strong anomalous scatterer atom at the Mo Kα wavelength, refinement of Flack's parameter was inconclusive. However, the absolute configuration of the molecule is known from the synthetic route.

### Crystal data

**Table:**

C_28_H_46_O_3_	*D*_x_ = 1.075 Mg m^−^^3^
*M**_r_* = 430.65	Melting point: 386 K
Monoclinic, *P*2_1_	Mo *K*α radiation, λ = 0.71073 Å
*a* = 7.5820 (1) Å	Cell parameters from 9561 reflections
*b* = 9.7487 (1) Å	θ = 2.4–21.0°
*c* = 17.8588 (2) Å	µ = 0.07 mm^−^^1^
β = 93.1792 (18)°	*T* = 293 K
*V* = 1318.00 (3) Å^3^	Prism, colourless
*Z* = 2	0.44 × 0.24 × 0.16 mm
*F*(000) = 476	

### Data collection

**Table:**

Bruker APEX CCD area-detector diffractometer	6194 independent reflections
Radiation source: fine-focus sealed tube	3551 reflections with *I* > 2σ(*I*)
graphite	*R*_int_ = 0.030
φ and ω scans	θ_max_ = 28.0°, θ_min_ = 2.3°
Absorption correction: multi-scan (*SADABS*; Sheldrick, 2000)	*h* = −9→9
*T*_min_ = 0.981, *T*_max_ = 0.989	*k* = −12→12
35109 measured reflections	*l* = −22→23

### Refinement

**Table:**

Refinement on *F*^2^	Secondary atom site location: difference Fourier map
Least-squares matrix: full	Hydrogen site location: inferred from neighbouring sites
*R*[*F*^2^ > 2σ(*F*^2^)] = 0.048	H-atom parameters constrained
*wR*(*F*^2^) = 0.138	*w* = 1/[σ^2^(*F*_o_^2^) + (0.0668*P*)^2^ + 0.1597*P*] where *P* = (*F*_o_^2^ + 2*F*_c_^2^)/3
*S* = 0.96	(Δ/σ)_max_ < 0.001
6194 reflections	Δρ_max_ = 0.23 e Å^−^^3^
286 parameters	Δρ_min_ = −0.14 e Å^−^^3^
1 restraint	Absolute structure: Flack (1983), 2846 Friedel pairs
Primary atom site location: structure-invariant direct methods	Flack parameter: −0.5 (15)

### Special details

**Table:**

Geometry. All e.s.d.'s (except the e.s.d. in the dihedral angle between two l.s. planes) are estimated using the full covariance matrix. The cell e.s.d.'s are taken into account individually in the estimation of e.s.d.'s in distances, angles and torsion angles; correlations between e.s.d.'s in cell parameters are only used when they are defined by crystal symmetry. An approximate (isotropic) treatment of cell e.s.d.'s is used for estimating e.s.d.'s involving l.s. planes.
Refinement. Refinement of *F*^2^ against ALL reflections. The weighted *R*-factor *wR* and goodness of fit *S* are based on *F*^2^, conventional *R*-factors *R* are based on *F*, with *F* set to zero for negative *F*^2^. The threshold expression of *F*^2^ > σ(*F*^2^) is used only for calculating *R*-factors(gt) *etc*. and is not relevant to the choice of reflections for refinement. *R*-factors based on *F*^2^ are statistically about twice as large as those based on *F*, and *R*- factors based on ALL data will be even larger.

### Fractional atomic coordinates and isotropic or equivalent isotropic displacement parameters (Å^2^)

**Table:**

	*x*	*y*	*z*	*U*_iso_*/*U*_eq_	
C9	0.9354 (3)	0.4824 (2)	0.25045 (11)	0.0496 (5)	
H9	0.8803	0.4012	0.2715	0.060*	
O56	0.59348 (19)	0.50504 (19)	0.26154 (8)	0.0663 (4)	
C10	0.8357 (3)	0.5092 (2)	0.17350 (11)	0.0537 (5)	
O3	0.3659 (2)	0.5251 (2)	0.03122 (9)	0.0795 (5)	
C12	1.1969 (3)	0.4357 (2)	0.33813 (13)	0.0561 (5)	
H12A	1.1475	0.3512	0.3566	0.067*	
H12B	1.3244	0.4265	0.3409	0.067*	
C13	1.1450 (3)	0.5549 (2)	0.38854 (11)	0.0492 (5)	
C7	0.8837 (3)	0.6045 (2)	0.29938 (12)	0.0517 (5)	
H7	0.9516	0.6841	0.2838	0.062*	
C14	0.9426 (3)	0.5733 (2)	0.37979 (11)	0.0521 (5)	
H14	0.8894	0.4854	0.3926	0.062*	
C20	1.3527 (3)	0.5455 (3)	0.51411 (12)	0.0631 (6)	
H20	1.3928	0.6398	0.5068	0.076*	
C17	1.1673 (3)	0.5317 (2)	0.47510 (11)	0.0563 (5)	
H17	1.1257	0.4386	0.4850	0.068*	
C11	1.1318 (3)	0.4564 (3)	0.25588 (12)	0.0597 (6)	
H11A	1.1929	0.5337	0.2350	0.072*	
H11B	1.1583	0.3754	0.2271	0.072*	
C6	0.6944 (3)	0.6292 (3)	0.27422 (13)	0.0621 (6)	
H6	0.6353	0.7138	0.2886	0.075*	
C5	0.6662 (3)	0.5752 (2)	0.19835 (13)	0.0573 (6)	
C3	0.4844 (3)	0.4820 (3)	0.09382 (13)	0.0672 (7)	
H3	0.4229	0.4183	0.1259	0.081*	
C1	0.7853 (3)	0.3819 (3)	0.12798 (13)	0.0670 (6)	
H1A	0.7392	0.3131	0.1610	0.080*	
H1B	0.8901	0.3444	0.1068	0.080*	
C18	1.2455 (3)	0.6852 (3)	0.36837 (14)	0.0664 (6)	
H18A	1.3703	0.6693	0.3755	0.100*	
H18B	1.2122	0.7592	0.4001	0.100*	
H18C	1.2169	0.7087	0.3169	0.100*	
C19	0.9377 (3)	0.6129 (3)	0.12773 (14)	0.0754 (7)	
H19A	0.8680	0.6373	0.0832	0.113*	
H19B	1.0469	0.5727	0.1141	0.113*	
H19C	0.9620	0.6936	0.1573	0.113*	
C21	1.4874 (4)	0.4498 (3)	0.48156 (17)	0.0861 (8)	
H21A	1.5950	0.4520	0.5125	0.129*	
H21B	1.5106	0.4791	0.4317	0.129*	
H21C	1.4416	0.3580	0.4799	0.129*	
C22	1.3466 (4)	0.5206 (3)	0.59896 (13)	0.0799 (8)	
H22A	1.2417	0.5644	0.6165	0.096*	
H22B	1.3356	0.4229	0.6076	0.096*	
C16	1.0325 (4)	0.6318 (3)	0.50653 (15)	0.0787 (8)	
H16A	1.0926	0.7120	0.5276	0.094*	
H16B	0.9696	0.5877	0.5458	0.094*	
C4	0.5304 (3)	0.6124 (3)	0.13705 (14)	0.0668 (7)	
H4A	0.5776	0.6805	0.1040	0.080*	
H4B	0.4258	0.6500	0.1584	0.080*	
O28	0.2305 (3)	0.3225 (3)	0.02880 (15)	0.1146 (8)	
C28	0.2462 (4)	0.4337 (4)	0.00463 (16)	0.0817 (8)	
C2	0.6466 (4)	0.4123 (3)	0.06445 (14)	0.0770 (8)	
H2A	0.6979	0.4712	0.0277	0.092*	
H2B	0.6114	0.3272	0.0397	0.092*	
C15	0.9018 (3)	0.6743 (3)	0.44114 (13)	0.0730 (7)	
H15A	0.7804	0.6656	0.4550	0.088*	
H15B	0.9225	0.7680	0.4256	0.088*	
C24	1.5074 (5)	0.5303 (4)	0.72649 (15)	0.1002 (10)	
H24A	1.3964	0.5577	0.7465	0.120*	
H24B	1.5132	0.4309	0.7288	0.120*	
C23	1.5041 (4)	0.5726 (4)	0.64467 (15)	0.0975 (10)	
H23A	1.5058	0.6720	0.6418	0.117*	
H23B	1.6103	0.5389	0.6230	0.117*	
C29	0.1328 (4)	0.4931 (4)	−0.05840 (16)	0.1031 (11)	
H29A	0.2018	0.5049	−0.1014	0.155*	
H29B	0.0879	0.5804	−0.0435	0.155*	
H29C	0.0361	0.4322	−0.0708	0.155*	
C25	1.6529 (5)	0.5861 (4)	0.77614 (16)	0.0989 (10)	
H25	1.6427	0.6863	0.7743	0.119*	
C26	1.8343 (5)	0.5507 (5)	0.7531 (2)	0.1229 (13)	
H26A	1.8534	0.4539	0.7588	0.184*	
H26B	1.9204	0.5996	0.7842	0.184*	
H26C	1.8452	0.5761	0.7016	0.184*	
C27	1.6293 (6)	0.5434 (6)	0.85708 (18)	0.1511 (18)	
H27A	1.5149	0.5719	0.8717	0.227*	
H27B	1.7190	0.5859	0.8893	0.227*	
H27C	1.6391	0.4455	0.8613	0.227*	

### Atomic displacement parameters (Å^2^)

**Table:**

	*U*^11^	*U*^22^	*U*^33^	*U*^12^	*U*^13^	*U*^23^
C9	0.0499 (11)	0.0536 (12)	0.0447 (11)	0.0052 (9)	−0.0024 (9)	−0.0030 (9)
O56	0.0528 (9)	0.0922 (12)	0.0533 (9)	−0.0007 (9)	−0.0012 (7)	−0.0018 (9)
C10	0.0518 (12)	0.0643 (14)	0.0442 (11)	0.0041 (11)	−0.0034 (9)	0.0013 (10)
O3	0.0679 (10)	0.1045 (14)	0.0629 (10)	−0.0054 (11)	−0.0248 (8)	0.0075 (11)
C12	0.0522 (12)	0.0576 (13)	0.0574 (13)	0.0101 (10)	−0.0069 (10)	0.0014 (11)
C13	0.0520 (12)	0.0456 (11)	0.0492 (11)	0.0046 (9)	−0.0056 (9)	0.0013 (9)
C7	0.0507 (12)	0.0520 (12)	0.0515 (12)	0.0073 (10)	−0.0058 (10)	−0.0058 (10)
C14	0.0537 (12)	0.0537 (12)	0.0480 (11)	0.0056 (10)	−0.0037 (9)	−0.0049 (10)
C20	0.0712 (15)	0.0587 (14)	0.0569 (13)	−0.0026 (12)	−0.0204 (11)	0.0066 (11)
C17	0.0622 (13)	0.0559 (13)	0.0495 (12)	−0.0048 (11)	−0.0094 (10)	0.0047 (11)
C11	0.0570 (13)	0.0693 (15)	0.0520 (12)	0.0161 (11)	−0.0043 (10)	−0.0080 (11)
C6	0.0560 (14)	0.0711 (15)	0.0580 (14)	0.0173 (12)	−0.0088 (11)	−0.0121 (12)
C5	0.0512 (12)	0.0651 (14)	0.0545 (13)	0.0078 (10)	−0.0074 (10)	0.0004 (11)
C3	0.0590 (14)	0.0877 (17)	0.0529 (13)	0.0013 (13)	−0.0150 (11)	0.0025 (13)
C1	0.0660 (15)	0.0805 (16)	0.0531 (14)	0.0097 (13)	−0.0100 (11)	−0.0131 (12)
C18	0.0660 (15)	0.0608 (14)	0.0704 (16)	−0.0063 (12)	−0.0149 (12)	0.0130 (12)
C19	0.0682 (16)	0.103 (2)	0.0546 (14)	−0.0009 (14)	−0.0025 (12)	0.0167 (14)
C21	0.0722 (17)	0.099 (2)	0.0840 (19)	0.0175 (15)	−0.0279 (14)	0.0004 (16)
C22	0.0922 (18)	0.0841 (19)	0.0598 (14)	−0.0182 (16)	−0.0270 (13)	0.0073 (14)
C16	0.0785 (18)	0.098 (2)	0.0579 (15)	0.0101 (15)	−0.0103 (13)	−0.0162 (14)
C4	0.0556 (14)	0.0803 (17)	0.0626 (14)	0.0086 (12)	−0.0129 (11)	0.0033 (13)
O28	0.127 (2)	0.0950 (16)	0.1150 (19)	−0.0149 (15)	−0.0555 (15)	−0.0021 (14)
C28	0.0705 (17)	0.107 (2)	0.0651 (17)	0.0073 (17)	−0.0157 (14)	−0.0145 (17)
C2	0.0760 (17)	0.096 (2)	0.0565 (15)	0.0054 (14)	−0.0159 (13)	−0.0154 (14)
C15	0.0675 (15)	0.0909 (18)	0.0591 (15)	0.0183 (14)	−0.0095 (12)	−0.0236 (14)
C24	0.121 (2)	0.113 (2)	0.0625 (16)	−0.030 (2)	−0.0267 (16)	0.0080 (18)
C23	0.108 (2)	0.112 (2)	0.0685 (17)	−0.0300 (19)	−0.0324 (15)	0.0102 (17)
C29	0.094 (2)	0.128 (3)	0.083 (2)	0.008 (2)	−0.0386 (16)	−0.007 (2)
C25	0.115 (3)	0.106 (2)	0.0716 (19)	−0.010 (2)	−0.0314 (18)	0.0022 (17)
C26	0.103 (3)	0.152 (3)	0.110 (3)	0.003 (3)	−0.026 (2)	−0.006 (3)
C27	0.167 (4)	0.214 (5)	0.068 (2)	−0.035 (4)	−0.033 (2)	0.011 (3)

### Geometric parameters (Å, °)

**Table:**

C9—C11	1.509 (3)	C18—H18B	0.9600
C9—C7	1.540 (3)	C18—H18C	0.9600
C9—C10	1.553 (3)	C19—H19A	0.9600
C9—H9	0.9800	C19—H19B	0.9600
O56—C6	1.443 (3)	C19—H19C	0.9600
O56—C5	1.454 (3)	C21—H21A	0.9600
C10—C1	1.521 (3)	C21—H21B	0.9600
C10—C5	1.525 (3)	C21—H21C	0.9600
C10—C19	1.535 (3)	C22—C23	1.497 (4)
O3—C28	1.340 (4)	C22—H22A	0.9700
O3—C3	1.457 (3)	C22—H22B	0.9700
C12—C13	1.534 (3)	C16—C15	1.546 (4)
C12—C11	1.537 (3)	C16—H16A	0.9700
C12—H12A	0.9700	C16—H16B	0.9700
C12—H12B	0.9700	C4—H4A	0.9700
C13—C18	1.535 (3)	C4—H4B	0.9700
C13—C14	1.544 (3)	O28—C28	1.175 (4)
C13—C17	1.562 (3)	C28—C29	1.495 (4)
C7—C6	1.500 (3)	C2—H2A	0.9700
C7—C14	1.511 (3)	C2—H2B	0.9700
C7—H7	0.9800	C15—H15A	0.9700
C14—C15	1.518 (3)	C15—H15B	0.9700
C14—H14	0.9800	C24—C25	1.480 (4)
C20—C21	1.522 (4)	C24—C23	1.517 (4)
C20—C22	1.538 (3)	C24—H24A	0.9700
C20—C17	1.540 (3)	C24—H24B	0.9700
C20—H20	0.9800	C23—H23A	0.9700
C17—C16	1.542 (4)	C23—H23B	0.9700
C17—H17	0.9800	C29—H29A	0.9600
C11—H11A	0.9700	C29—H29B	0.9600
C11—H11B	0.9700	C29—H29C	0.9600
C6—C5	1.458 (3)	C25—C26	1.497 (5)
C6—H6	0.9800	C25—C27	1.524 (4)
C5—C4	1.505 (3)	C25—H25	0.9800
C3—C4	1.517 (4)	C26—H26A	0.9600
C3—C2	1.524 (4)	C26—H26B	0.9600
C3—H3	0.9800	C26—H26C	0.9600
C1—C2	1.533 (3)	C27—H27A	0.9600
C1—H1A	0.9700	C27—H27B	0.9600
C1—H1B	0.9700	C27—H27C	0.9600
C18—H18A	0.9600		
			
C11—C9—C7	112.00 (17)	H18A—C18—H18C	109.5
C11—C9—C10	121.01 (17)	H18B—C18—H18C	109.5
C7—C9—C10	104.22 (16)	C10—C19—H19A	109.5
C11—C9—H9	106.2	C10—C19—H19B	109.5
C7—C9—H9	106.2	H19A—C19—H19B	109.5
C10—C9—H9	106.2	C10—C19—H19C	109.5
C6—O56—C5	60.45 (15)	H19A—C19—H19C	109.5
C1—C10—C5	107.96 (18)	H19B—C19—H19C	109.5
C1—C10—C19	111.84 (19)	C20—C21—H21A	109.5
C5—C10—C19	109.6 (2)	C20—C21—H21B	109.5
C1—C10—C9	115.54 (19)	H21A—C21—H21B	109.5
C5—C10—C9	100.95 (16)	C20—C21—H21C	109.5
C19—C10—C9	110.31 (18)	H21A—C21—H21C	109.5
C28—O3—C3	117.2 (2)	H21B—C21—H21C	109.5
C13—C12—C11	112.47 (17)	C23—C22—C20	114.7 (2)
C13—C12—H12A	109.1	C23—C22—H22A	108.6
C11—C12—H12A	109.1	C20—C22—H22A	108.6
C13—C12—H12B	109.1	C23—C22—H22B	108.6
C11—C12—H12B	109.1	C20—C22—H22B	108.6
H12A—C12—H12B	107.8	H22A—C22—H22B	107.6
C12—C13—C18	110.04 (18)	C17—C16—C15	107.76 (19)
C12—C13—C14	108.29 (17)	C17—C16—H16A	110.2
C18—C13—C14	112.56 (17)	C15—C16—H16A	110.2
C12—C13—C17	116.99 (17)	C17—C16—H16B	110.2
C18—C13—C17	108.93 (18)	C15—C16—H16B	110.2
C14—C13—C17	99.73 (15)	H16A—C16—H16B	108.5
C6—C7—C14	123.00 (19)	C5—C4—C3	107.3 (2)
C6—C7—C9	102.89 (17)	C5—C4—H4A	110.3
C14—C7—C9	108.20 (16)	C3—C4—H4A	110.3
C6—C7—H7	107.3	C5—C4—H4B	110.3
C14—C7—H7	107.3	C3—C4—H4B	110.3
C9—C7—H7	107.3	H4A—C4—H4B	108.5
C7—C14—C15	119.57 (18)	O28—C28—O3	124.3 (3)
C7—C14—C13	111.05 (16)	O28—C28—C29	124.7 (3)
C15—C14—C13	104.11 (17)	O3—C28—C29	111.0 (3)
C7—C14—H14	107.2	C3—C2—C1	111.4 (2)
C15—C14—H14	107.2	C3—C2—H2A	109.3
C13—C14—H14	107.2	C1—C2—H2A	109.3
C21—C20—C22	109.7 (2)	C3—C2—H2B	109.3
C21—C20—C17	112.9 (2)	C1—C2—H2B	109.3
C22—C20—C17	110.8 (2)	H2A—C2—H2B	108.0
C21—C20—H20	107.7	C14—C15—C16	102.9 (2)
C22—C20—H20	107.7	C14—C15—H15A	111.2
C17—C20—H20	107.7	C16—C15—H15A	111.2
C20—C17—C16	112.78 (19)	C14—C15—H15B	111.2
C20—C17—C13	118.75 (18)	C16—C15—H15B	111.2
C16—C17—C13	103.37 (17)	H15A—C15—H15B	109.1
C20—C17—H17	107.1	C25—C24—C23	116.7 (3)
C16—C17—H17	107.1	C25—C24—H24A	108.1
C13—C17—H17	107.1	C23—C24—H24A	108.1
C9—C11—C12	110.30 (18)	C25—C24—H24B	108.1
C9—C11—H11A	109.6	C23—C24—H24B	108.1
C12—C11—H11A	109.6	H24A—C24—H24B	107.3
C9—C11—H11B	109.6	C22—C23—C24	113.8 (3)
C12—C11—H11B	109.6	C22—C23—H23A	108.8
H11A—C11—H11B	108.1	C24—C23—H23A	108.8
O56—C6—C5	60.15 (14)	C22—C23—H23B	108.8
O56—C6—C7	113.77 (18)	C24—C23—H23B	108.8
C5—C6—C7	108.02 (18)	H23A—C23—H23B	107.7
O56—C6—H6	120.1	C28—C29—H29A	109.5
C5—C6—H6	120.1	C28—C29—H29B	109.5
C7—C6—H6	120.1	H29A—C29—H29B	109.5
O56—C5—C6	59.41 (15)	C28—C29—H29C	109.5
O56—C5—C4	113.85 (19)	H29A—C29—H29C	109.5
C6—C5—C4	130.4 (2)	H29B—C29—H29C	109.5
O56—C5—C10	112.92 (18)	C24—C25—C26	114.6 (3)
C6—C5—C10	109.87 (17)	C24—C25—C27	110.2 (3)
C4—C5—C10	116.33 (19)	C26—C25—C27	110.8 (3)
O3—C3—C4	105.3 (2)	C24—C25—H25	106.9
O3—C3—C2	109.85 (19)	C26—C25—H25	106.9
C4—C3—C2	112.4 (2)	C27—C25—H25	106.9
O3—C3—H3	109.7	C25—C26—H26A	109.5
C4—C3—H3	109.7	C25—C26—H26B	109.5
C2—C3—H3	109.7	H26A—C26—H26B	109.5
C10—C1—C2	112.3 (2)	C25—C26—H26C	109.5
C10—C1—H1A	109.1	H26A—C26—H26C	109.5
C2—C1—H1A	109.1	H26B—C26—H26C	109.5
C10—C1—H1B	109.1	C25—C27—H27A	109.5
C2—C1—H1B	109.1	C25—C27—H27B	109.5
H1A—C1—H1B	107.9	H27A—C27—H27B	109.5
C13—C18—H18A	109.5	C25—C27—H27C	109.5
C13—C18—H18B	109.5	H27A—C27—H27C	109.5
H18A—C18—H18B	109.5	H27B—C27—H27C	109.5
C13—C18—H18C	109.5		
			
C11—C9—C10—C1	82.1 (3)	C6—O56—C5—C10	100.3 (2)
C7—C9—C10—C1	−150.83 (18)	C7—C6—C5—O56	107.6 (2)
C11—C9—C10—C5	−161.8 (2)	O56—C6—C5—C4	96.5 (3)
C7—C9—C10—C5	−34.7 (2)	C7—C6—C5—C4	−155.9 (2)
C11—C9—C10—C19	−46.0 (3)	O56—C6—C5—C10	−105.5 (2)
C7—C9—C10—C19	81.1 (2)	C7—C6—C5—C10	2.1 (3)
C11—C12—C13—C18	−67.9 (2)	C1—C10—C5—O56	77.9 (2)
C11—C12—C13—C14	55.6 (2)	C19—C10—C5—O56	−160.12 (18)
C11—C12—C13—C17	167.16 (19)	C9—C10—C5—O56	−43.8 (2)
C11—C9—C7—C6	169.22 (19)	C1—C10—C5—C6	142.1 (2)
C10—C9—C7—C6	36.7 (2)	C19—C10—C5—C6	−95.9 (2)
C11—C9—C7—C14	−59.2 (2)	C9—C10—C5—C6	20.5 (2)
C10—C9—C7—C14	168.28 (17)	C1—C10—C5—C4	−56.5 (3)
C6—C7—C14—C15	−58.0 (3)	C19—C10—C5—C4	65.5 (3)
C9—C7—C14—C15	−177.5 (2)	C9—C10—C5—C4	−178.1 (2)
C6—C7—C14—C13	−179.2 (2)	C28—O3—C3—C4	−150.8 (2)
C9—C7—C14—C13	61.2 (2)	C28—O3—C3—C2	87.9 (3)
C12—C13—C14—C7	−59.8 (2)	C5—C10—C1—C2	52.3 (3)
C18—C13—C14—C7	62.1 (2)	C19—C10—C1—C2	−68.3 (3)
C17—C13—C14—C7	177.40 (17)	C9—C10—C1—C2	164.4 (2)
C12—C13—C14—C15	170.26 (19)	C21—C20—C22—C23	72.5 (3)
C18—C13—C14—C15	−67.9 (2)	C17—C20—C22—C23	−162.2 (3)
C17—C13—C14—C15	47.5 (2)	C20—C17—C16—C15	146.4 (2)
C21—C20—C17—C16	−178.2 (2)	C13—C17—C16—C15	16.9 (3)
C22—C20—C17—C16	58.2 (3)	O56—C5—C4—C3	−76.4 (3)
C21—C20—C17—C13	−57.1 (3)	C6—C5—C4—C3	−145.6 (3)
C22—C20—C17—C13	179.3 (2)	C10—C5—C4—C3	57.5 (3)
C12—C13—C17—C20	79.4 (3)	O3—C3—C4—C5	−174.84 (19)
C18—C13—C17—C20	−46.1 (2)	C2—C3—C4—C5	−55.2 (3)
C14—C13—C17—C20	−164.20 (19)	C3—O3—C28—O28	0.5 (4)
C12—C13—C17—C16	−154.9 (2)	C3—O3—C28—C29	179.1 (2)
C18—C13—C17—C16	79.6 (2)	O3—C3—C2—C1	172.8 (2)
C14—C13—C17—C16	−38.4 (2)	C4—C3—C2—C1	55.9 (3)
C7—C9—C11—C12	55.5 (2)	C10—C1—C2—C3	−54.3 (3)
C10—C9—C11—C12	179.0 (2)	C7—C14—C15—C16	−161.8 (2)
C13—C12—C11—C9	−54.3 (3)	C13—C14—C15—C16	−37.1 (3)
C5—O56—C6—C7	−97.9 (2)	C17—C16—C15—C14	12.1 (3)
C14—C7—C6—O56	−81.5 (3)	C20—C22—C23—C24	−172.1 (3)
C9—C7—C6—O56	40.5 (2)	C25—C24—C23—C22	−175.9 (3)
C14—C7—C6—C5	−146.1 (2)	C23—C24—C25—C26	−58.7 (5)
C9—C7—C6—C5	−24.1 (2)	C23—C24—C25—C27	175.5 (4)
C6—O56—C5—C4	−124.2 (2)	C19—C10—C13—C18	12.68 (18)
